# Adoptive immunotherapy shows encouraging benefit on non-small cell lung cancer: a systematic review and meta-analysis

**DOI:** 10.18632/oncotarget.19373

**Published:** 2017-07-19

**Authors:** Binghao Zhao, Wenxiong Zhang, Dongliang Yu, Jianjun Xu, Yiping Wei

**Affiliations:** ^1^ Department of Thoracic Surgery, The Second Affiliated Hospital of Nanchang University, Nanchang, China

**Keywords:** non-small cell lung cancer, adoptive immunotherapy, meta-analysis

## Abstract

Although adoptive immunotherapy (AIT) is a novel emerging target treatment for non-small cell lung cancer (NSCLC), its actual efficacy remains controversial. In this meta-analysis, we aimed to evaluate the efficacy of AIT for NSCLC. We systematically searched PubMed, the Cochrane Library, EMBASE, Medline, and Web of Science for relevant parallel randomized controlled trials (RCTs) and high-quality observation studies of AIT without any language restrictions. Two investigators reviewed all the texts and extracted information regarding overall survival rate (OS), progression-free survival rate (PFS), objective response rate (ORR), and disease control rate (DCR) from eligible studies; sensitivity analyses and subgroup analyses were also conducted to reduce heterogeneity

Of 319 suitable studies, 15 studies (13 RCTs and 2 observation studies) involving 1684 patients were finally included. Compared to the Control therapy (CT) group, the AIT group exhibited better 1-year OS (*P* = 0.001), 2-year OS (*P* < 0.001), 3-year OS (*P* < 0.001), 5-year OS (*P* = 0.032), 1-year PFS (*P* < 0.001), and 2-year PFS (*P* = 0.029). The difference in the ORR (*P* = 0.293) and DCR (*P* = 0.123) was not significant between the groups. The subgroup analysis showed that DC/CIK did more benefit to NSCLC patients than LAK and the cycles not associated with AIT efficacy.

AIT can significantly improve the OS and PFS with acceptable toxicity for NSCLC. Nevertheless, further multicenter studies are needed to confirm our conclusion and determine which adoptive immunotherapy is associated with the greatest efficacy.

## INTRODUCTION

Non-small cell lung cancer (NSCLC) is one of the most common malignant tumors and accounts for 80–85% of all cases of lung cancer [1]. In the United States, the mortality due to lung cancer is the highest among all cancers. Although marked progress has been made in surgery, radiotherapy, and chemotherapy for NSCLC, the 5-year overall survival rate (OS) remains unsatisfactory (approximately 15%) [2]. Hence, researchers are currently seeking to improve the long-term OS rate of NSCLC after surgery or advanced NSCLC. Immunotherapy plays an effective and beneficial role in several malignant tumors that activate our immune system to produce an anti-tumor effect. Advanced research in immunotherapy led to the development of adoptive immunotherapy (AIT), which has major benefits and potential for further enhancement. AIT involves the transfusion of activated lymphocytes or lymphocyte products to cancer patients to enhance the patient's immunity and anti-tumor ability [3]. There are various types of AIT methods and marked heterogeneity in the effector cells used, including natural killer cells (NK), cytotoxic T lymphocytes (CTL), lymphokine-activated killer cells (LAK), cytokine-induced killer cells (CIK), dendritic cells (DC), and tumor infiltrating lymphocytes (TIL) [4]. However, the efficacy of AIT remains controversial. Therefore, in the present study, we systematically searched parallel randomized controlled trials (RCTs) and conducted a systematic review and meta-analysis to evaluate the efficacy of AIT for NSCLC patients, in order to provide an objective reference for clinical decision making.

## RESULTS

**Figure 1:**
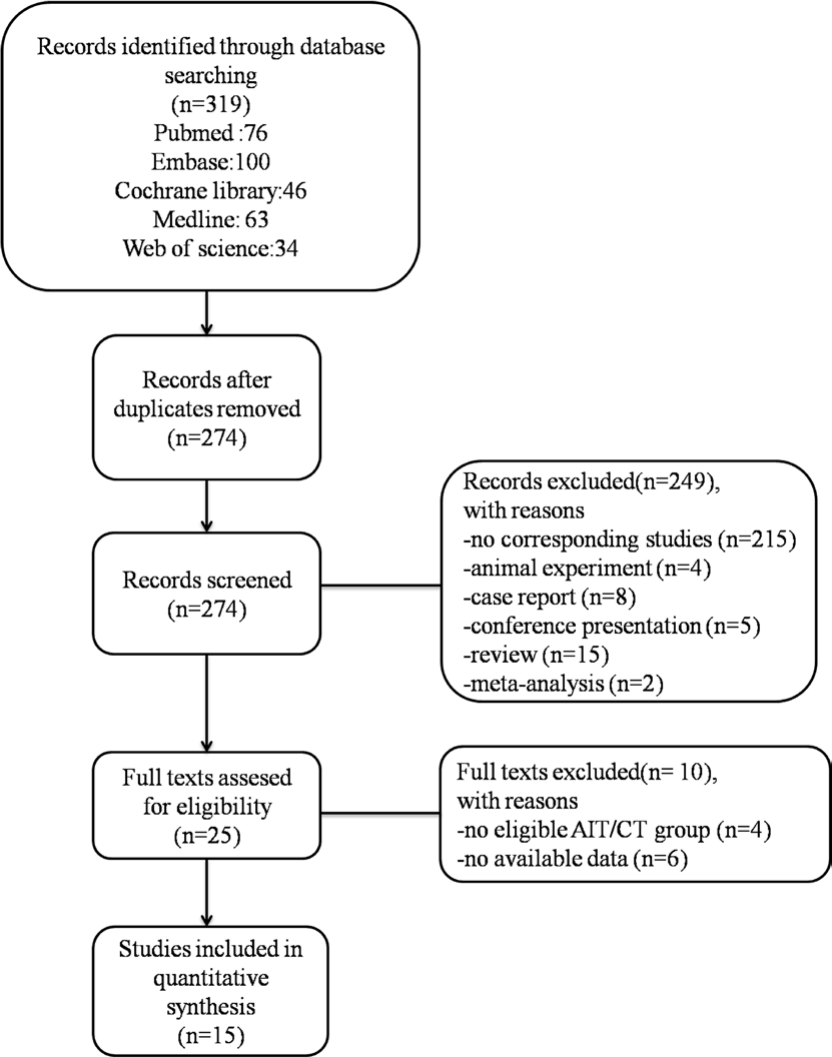
Study selection process

Of the 319 studies searched in this meta-analysis, we finally included 15 studies [5–19]; the selection process is described in Figure 1.

The characteristics of the 15 included studies (1684 patients) are described in Table 1, and the outcome data are presented in Table 2. Of the 15 studies, 13 [1–7, 9, 11–15] were parallel RCTs and the other 2 [8, 10] were prospective cohort studies. As for pre-treatment, 7 [5–8, 10, 16, 18] studies included patients with pre-surgery, 2 [17, 19] studies included patients with chemotherapy, 1 [9] study included patients with surgery or chemotherapy or radiotherapy. With regard to the AIT regimen, 3 studies [5, 7, 8] used LAK plus IL-2 (or rIL-2), 7 studies [10–11, 14–19] used DC/CIK, 2 studies [9, 13] used CIK alone, 2 studies [12, 18] used activated killer T cells (AKT) alone, and 1 study [6] used TIL alone. The regimen involved > 4 cycles in 8 studies [5–7, 9, 12, 13, 18, 19] and ≤ 4 cycles in 4 studies [10, 11, 14, 16]. The precise cycle was not described in 1 study [8] and we could not determine whether the cycles were more than, equal to, or less than 4 in 2 studies [15, 17]. All the studies included Asian participants, except for 1 study [6].

**Table 1:** Characteristics of included studies

	Study	Region	Tumor Stage	Sample size (I/C)	Interventions	Duration	Outcome
1995	Kimura [5]	Japan	I–IV	49	LAK+ IL-2 Immunotherapy	mean 6.4 cycles	①②③④
				52	Radiation+ Chemotherapy (cisplatin+ vindesine+ mitomycin)		
1996	Ratto[6]	Italy	II, IIIa, IIIb	56	TIL+rIL-2 Immunotherapy	mean 6 cycles	①②③
				57	Chemotherapy (vinblastine+ cisplatin+ 50 gray in 25 fractions)		
1997	Kimura [7]	Japan	I–IV	82	LAK+IL-2 Immunotherapy	mean 6 cycles	①②③④
				88	no adjuvant therapy		
1999	Yano [8]	Japan	I–IV	19	LAK+rIL-2 Immunotherapy	not mentioned	①②③④
				21	no adjuvant therapy		
2008	Wu [9]	China	IIIa, IIIb, IV	29	CIK Immunotherapy+Chemotherapy	mean 6 cycles	①②⑤⑦⑧
				30	Chemotherapy (docetaxel+ cisplatin)		
2009	Li [10]	China	I, II, IIIa	42	DC/CIK Immunotherapy+ Chemotherapy	mean 4 cycles	①②③⑤⑥
				42	Chemotherapy ( navelbine+ cisplatin)		
2011	Zhong [11]	China	IIIb, IV	14	DC/CIL Immunotherapy+ Chemotherapy	mean 4 cycles	①②③
				14	Chemotherapy (platinum)		
2012	Iwai [12]	Japan	IIIb, IV	132	AKT Immunotherapy with simultaneous	4–6 cycles	①②③④
				207	Chemotherapy (platinum-containing+ anticancer drugs)		
2012	Li [13]	China	I–IV	87	CIK Immunotherapy+ Chemotherapy	total 6 cycles	①②③④⑤⑥
				87	Chemotherapy (paclitaxel gemcitabine navelbine+ cisplatin)		
2013	Yang [14]	China	IIIa, IIIb, IV	61	DC/CIK Immunotherapy+ Chemotherapy	mean 4 cycles	①②⑦⑧
				61	Chemotherapy (Navelbin+ Cisplatin)		
2014	Zhong [15]	China	IIIb, IV	30	DC/CIK Immunotherapy+ Chemotherapy	2–6 cycles	①②③⑦⑧
				30	DC/CIK+Chemotherapy (navelbine+ platinum)		
2014	Zhao [16]	China	IIIa	79	DC/CIK Immunotherapy+ Chemotherapy (gemcitabine+ platinum)	mean 4 cycles	①②③
				78	Chemotherapy (gemcitabine+ platinum)		
2014	Shi [17]	China	IIIb, IV	28	DC/CIK Immunotherapy+ Chemotherapy (erlotinib)	at least 2 cycles	①
				28	Chemotherapy (erlotinib)		
2015	Kimura [18]	Japan	Ib–IV	50	AKT/DC Immunotherapy+ Chemotherapy	total 12–15 cycles	①②④⑤⑥
				51	Chemotherapy (platinum)		
2016	Zhang [19]	China	IIIa, IIIb, IV	21	DC/CIK Immunotherapy+ radiotherapy	mean 6 cycles	①⑤⑦⑧
				61	radiotherapy		

Abbreviations: LAK: lymphokine-activated killer T cells; TIL: tumor infiltrating lymphocyte; CIK: cytokine-induced killer cells; DC: dendritic cells; AKT: activated killer T cells; (r)IL-2: (recomibiant)Interleukin-2. ①: 1-year overall survival rate (OS); ②: 2-year OS; ③: 3-year OS; ④: 5-year OS; ⑤: 1-year progression-free-survival rate (PFS); ⑥: 2-year PFS; ⑦: Objective response rate (ORR); ⑧: Disease control rate (DCR).

**Table 2:** Original data extracted from included studies

Study		Sample size (I/C)	1-year OS (Pts, %)	2-year OS (Pts, %)	3-year OS (Pts, %)	5-year OS (Pts, %)	1-year PFS (Pts, %)	2-year PFS (Pts, %)	ORR (Pts, %)	DCR (Pts, %)
1995	Kimura [5]	49	43, 87.8%	32, 65.3%	23, 46.9%	21, 42.9%	/	/	/	/
		52	39, 75.0%	22, 42.3%	15, 28.8%	9, 17.3%	/	/	/	/
1996	Ratto[6]	56	35, 62.5%	20, 35.7%	14, 25.0%	/	/	/	/	/
		57	25, 43.9%	12, 21.1%	7, 12.3%	/	/	/	/	/
1997	Kimura [7]	82	73, 89.0%	66, 75.0%	47, 57.3%	45, 54.4%	/	/	/	/
		88	66, 75.0%	46, 52.3%	35, 39.8%	16, 33.4%	/	/	/	/
1999	Yano [8]	19	17, 89.5%	15, 78.9%	14, 73.7%	13, 68.4%	/	/	/	/
		21	20, 95.2%	18, 85.7%	16, 76.2%	13, 61.9%	/	/	/	/
2008	Wu [9]	29	20, 69.0%	8, 27.6%	/	/	2, 6.9%	/	13, 44.8%	26, 89.7%
		30	14, 46.7%	4, 13.3%	/	/	1, 3.3%	/	13, 43.3%	20, 65.5%
2009	Li [10]	42	41, 97.6%	40, 94.7%	40, 94.7%	/	41, 97.6%	32, 76.2%	/	/
		42	35, 83.3%	33, 78.8%	32, 76.2%	/	34, 81.0%	27, 64.3%	/	/
2011	Zhong [11]	14	9, 64.3%	7, 49.8%	3, 21.4%	/	/	/	/	/
		14	6, 42.8%	4, 28.5%	1, 7.1%	/	/	/	/	/
2012	Iwai [12]	132	95, 72.0%	55, 41.9%	32, 24.2%	13, 9.8%	/	/	/	/
		207	124, 60.0%	77, 37.1%	50, 24.2%	33, 16.0%	/	/	/	/
2012	Li [13]	87	78, 89.7%	65, 74.7%	52, 59.8%	26, 29.9%	63, 72.4%	45, 51.7%	/	/
		87	58, 66.7%	38, 43.7%	34, 39.1%	16, 18.4%	44, 50.6%	32, 36.8%	/	/
2013	Yang [14]	61	35, 57.2%	16, 27.0%	/	/	/	/	11, 18.0%	42, 68.9%
		61	23, 37.3%	7, 10.1%	/	/	/	/	10, 16.4%	30, 49.2%
2014	Zhong [15]	30	19, 63.3%	9, 30.0%	7, 23.3%	/	/	/	5, 16.7%	21, 70.0%
		30	18, 60.0%	7, 21.7%	4, 13.3%	/	/	/	6, 20.0%	21, 70.0%
2014	Zhao [16]	79	73, 92.4%	55, 69.6%	46, 58.2%	/	/	/	/	/
		78	62, 79.5%	43, 55.1%	29, 37.2%	/	/	/	/	/
2014	Shi [17]	28	6, 21.4%	/	/	/	/	/	/	/
		26	4, 15.4%	/	/	/	/	/	/	/
2015	Kimura [18]	50	49, 98.0%	47, 94.0%	/	41, 81.4%	45, 90.0%	35, 70.0%	/	/
		51	48, 94.1%	34, 66.7%	/	25, 48.3%	32, 62.7%	15, 29.4%	/	/
2016	Zhang [19]	21	16, 76.2%	/	/	/	8, 38.1%	/	10, 47.6%	19, 90.5%
		61	36, 59.0%	/	/	/	12, 19.7%	/	15, 24.6%	54, 88.5%

The risk and bias assessments [20] of the included RCTs and observation studies are described in Table 3.

Table 3Quality criteria and risk of bias

**Table:** 

	Low risk of bias No. (%)	High risk of bias No. (%)	Unclear risk of bias No. (%)
**Power calculation of adoptive immunotherapy therapy**	15 (100)	0 (0)	0 (0)
**No conflicts of interest, funding**	11 (73.3)	0 (0)	4 (26.7)
**Risk of bias assessments^a^**			
Random sequence generation	9 (69.2)	2 (15.4)	2 (15.4)
Allocation concealment	7 (53.8)	0 (0)	6 (46.2)
Blinding of participants and personnel	5 (38.5)	2 (15.4)	6 (46.1)
Blinding of outcome assessment	8 (61.5)	0 (0)	5 (38.5)
Incomplete outcome data	11 (84.6)	0 (0)	2 (15.4)
Selective reporting	12 (92.3)	0 (0)	1 (7.7)
Other bias	3 (23.1)	0 (0)	10 (76.9)

^a^Risk of bias assessments was base on “Cochrane risk of bias tool”.

**Table:** 

	Study^b^	Selection				Comparability	Outcome			Total score
		Exposed cohort	Nonexposed cohort	Ascertainment of exposure	Outcome of interest		Assessment of outcome	Length of follow-up	Adequacy of follow-up	
2012	Iwai	^*^	^*^	^*^	^*^	^**^	^*^	^*^	^*^	9
2013	Yang	^*^	^*^	^*^	^*^	^**^	^*^			7

^b^Risk of bias assessments was base on “the Newcastle-Ottawa Scale (NOS)”.

### 1-year OS

**Figure 2:**
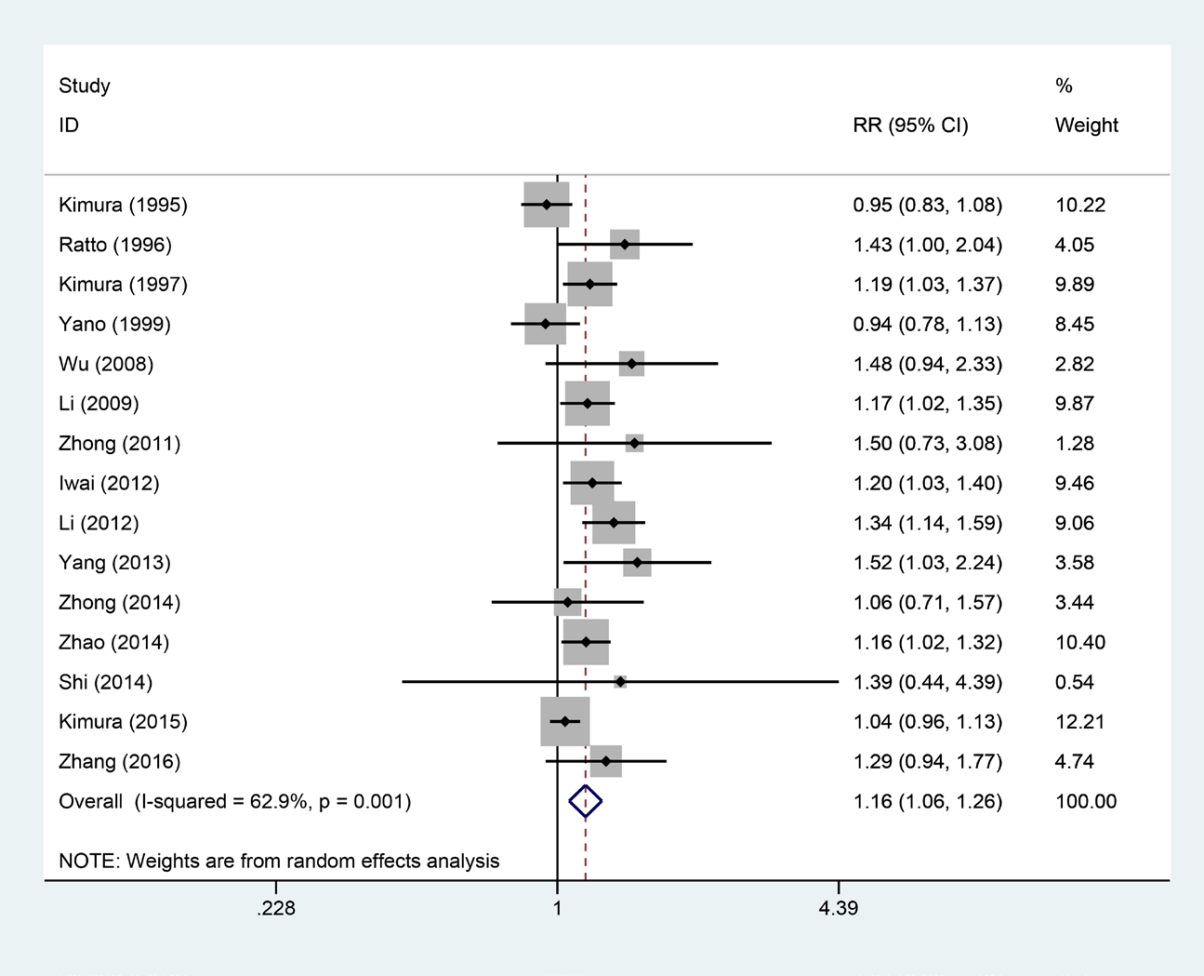
One-year OS between the AIT and CT groups

Fifteen studies [5–19], involving 1684 patients (AIT group: *n* = 779; Control therapy [CT] group: *n* = 905), were included in this analysis (Figure 2). High heterogeneity was observed (*P* = 0.001, I^2^ = 62.9%), and a random-effects model was used. We found that the 1-year OS was better in the AIT group than in the CT group (95% confidence interval [CI], 1.06–1.26; *P* = 0.001).

### 2-year OS

**Figure 3:**
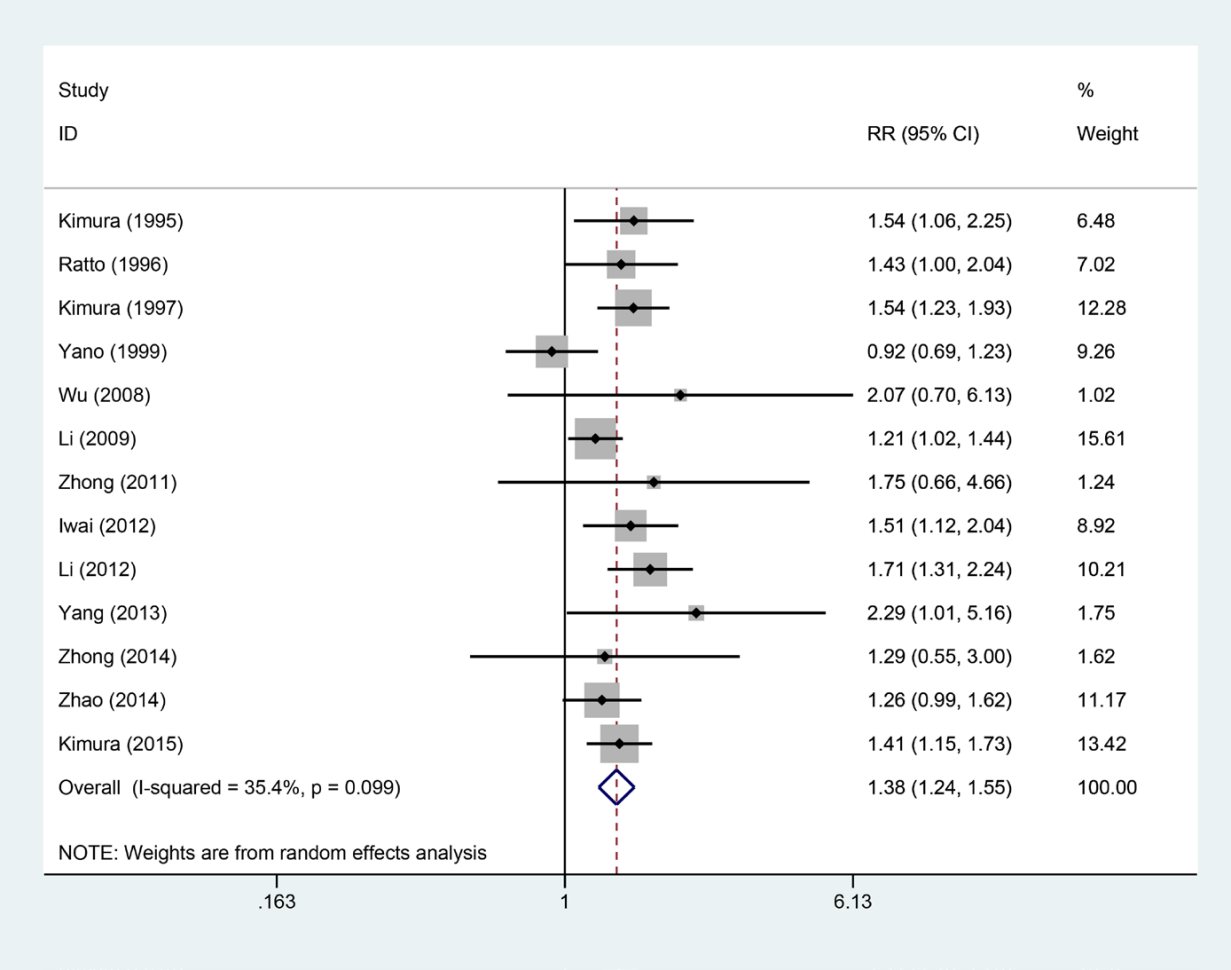
Two-year OS between the AIT and CT groups

Thirteen studies [5–16, 18], involving 1548 patients (AIT group: *n* = 730; CT group: *n* = 818), were included in this analysis (Figure 3). High heterogeneity was observed (*P* = 0.099, I^2^ = 35.4%), and a random-effects model was used. We found that the 2-year OS was better in the AIT group than in the CT group (95% CI, 1.24–1.55; *P* < 0.001).

### 3-year OS

**Figure 4:**
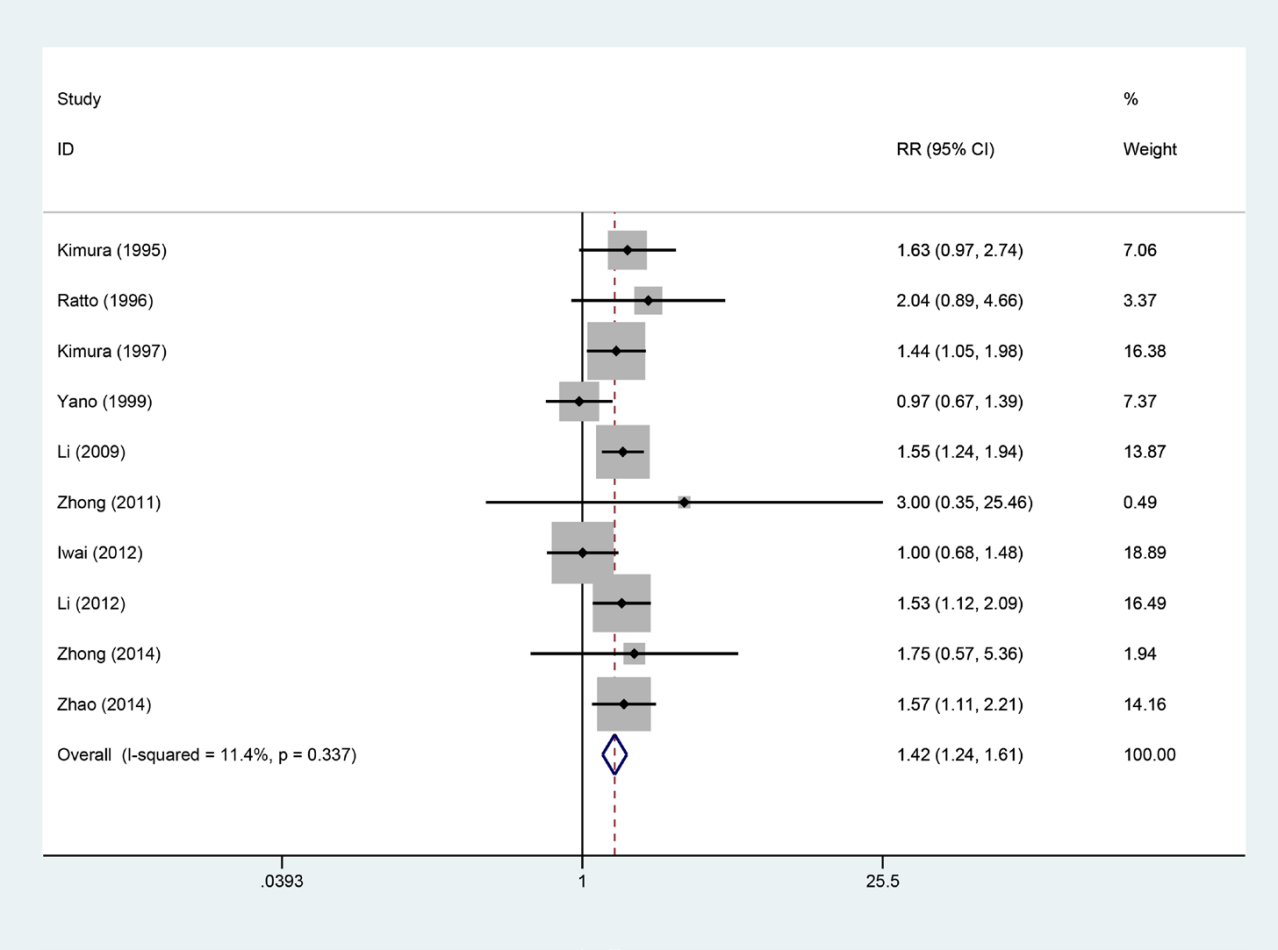
Three-year OS between the AIT and CT groups

Ten studies [5–8, 10–13, 15, 16], involving 1266 patients (AIT group: *n* = 590; CT group: *n* = 676), were included in this analysis (Figure 4). Low heterogeneity was observed (*P* = 0.337, I^2^ = 11.4%), and a fixed-effects model was used. We found that the 3-year OS was better in the AIT group than in the CT group (95% CI, 1.24–1.61; *P* < 0.001).

### 5-year OS

**Figure 5:**
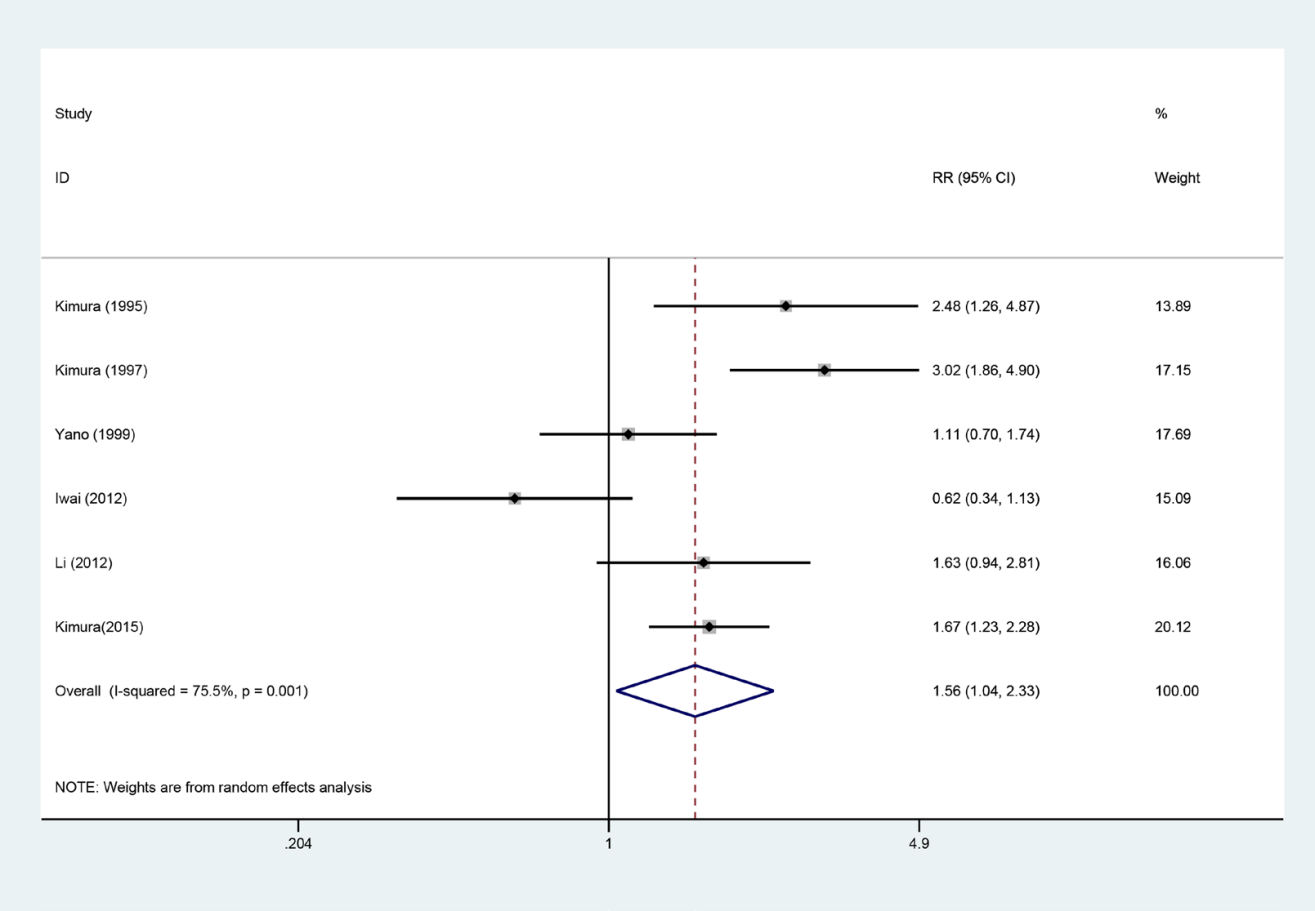
Five-year OS between the AIT and CT groups

Six studies [5, 7, 8, 12, 13, 18], involving 925 patients (AIT group: *n* = 419; CT group: *n* = 506), were included in this analysis (Figure 5). High heterogeneity was observed (*P* = 0.01, I^2^ = 75.5%), and a random-effects model was used. We found that the 5-year OS was better in the AIT group than in the CT group (95% CI, 1.04–2.33; *P* = 0.032).

### 1-year PFS

**Figure 6:**
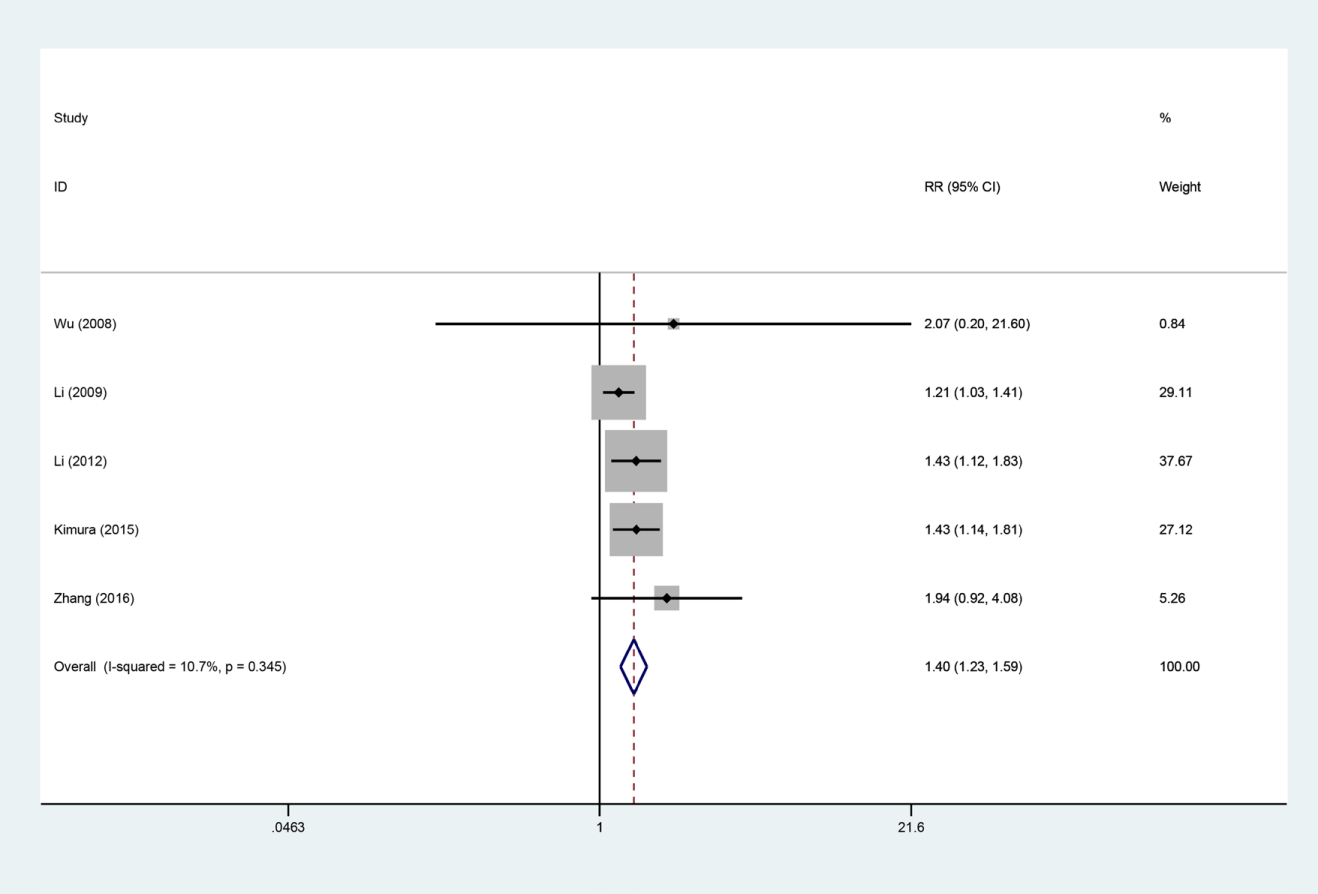
One-year PFS between the AIT and CT groups

Five studies [9, 10, 13, 18, 19], involving 519 patients (AIT group: *n* = 229; CT group: *n* = 271), were included in this analysis (Figure 6). Low heterogeneity was observed (*P* = 0.345, I^2^ = 10.7%), and a fixed-effects model was used. We found that the 1-year progression-free survival rate (PFS) was better in the AIT group than in the CT group (95% CI, 1.23–1.59; *P* < 0.001).

### 2-year PFS

**Figure 7:**
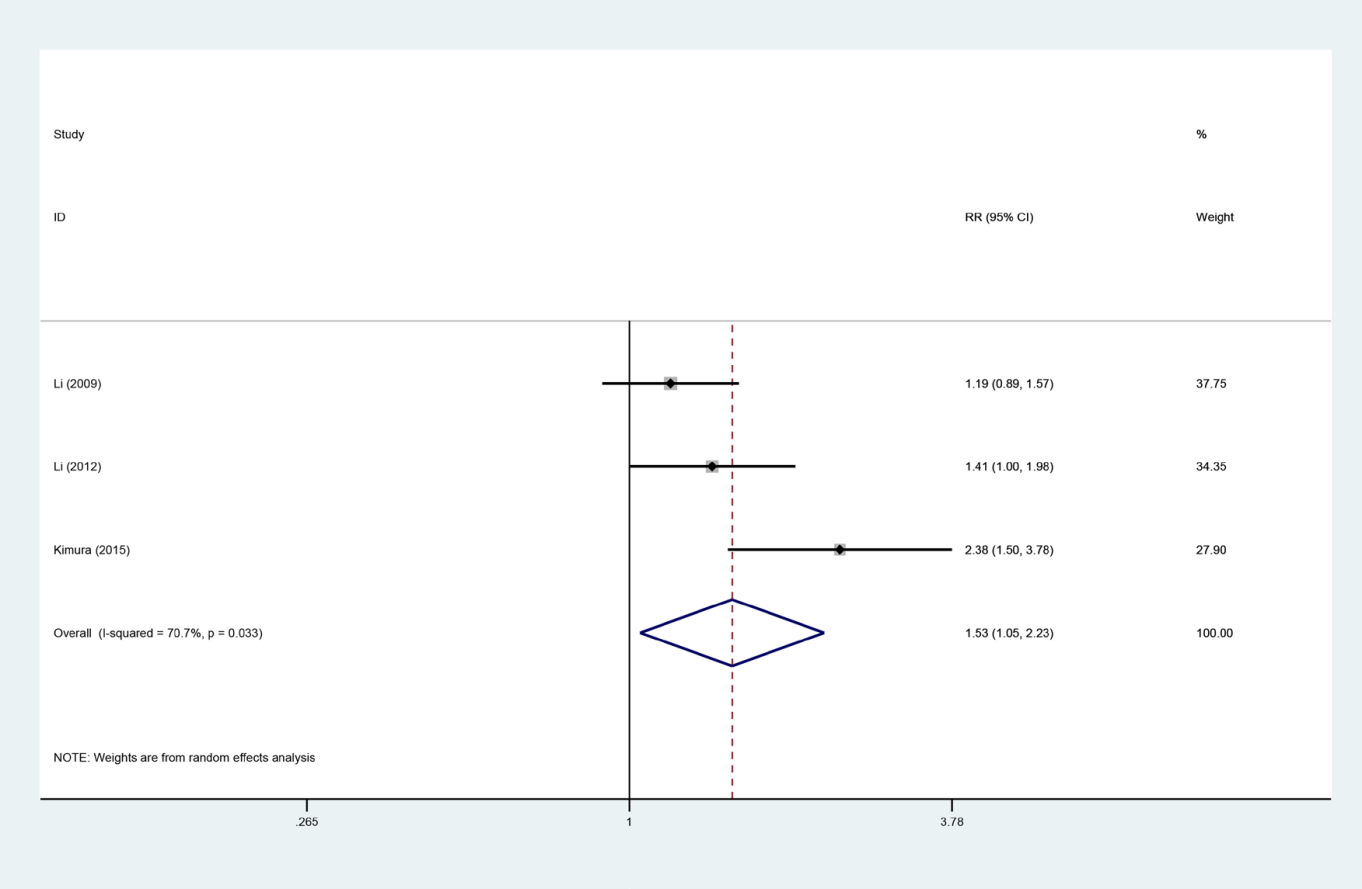
Two-year PFS between the AIT and CT groups

Three studies [10, 13, 18], involving 353 patients (AIT group: *n* = 173; CT group: *n* = 180), were included in this analysis (Figure 7). High heterogeneity was observed (*P* = 0.033, I^2^ = 70.7%), and a random-effects model was used. We found that the 2-year PFS was better in the AIT group than in the CT group (95% CI, 1.05–2.23; *P* = 0.029).

### ORR

**Figure 8:**
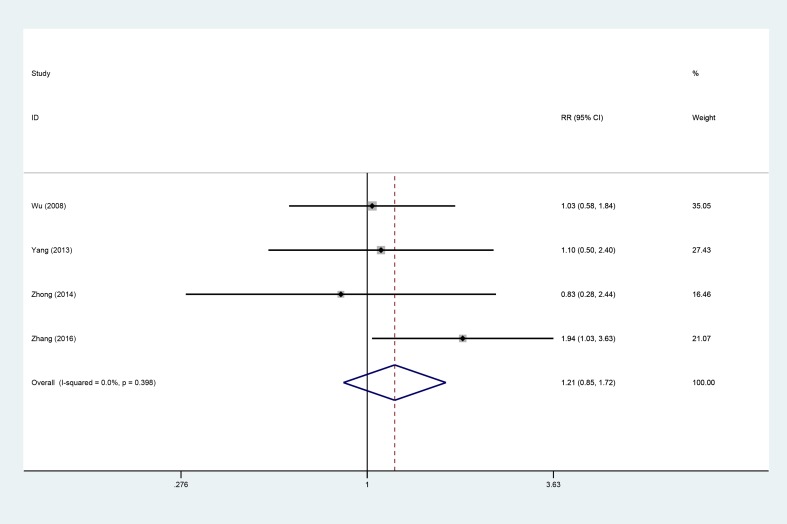
ORR between the AIT and CT groups

Four studies [9, 14, 15, 19], involving 323 patients (AIT group: *n* = 141; CT group: *n* = 182), were included in this analysis (Figure 8). Low heterogeneity was observed (*P* = 0.398, I^2^ = 0%), and a fixed-effects model was used. The objective response rate (ORR) did not significantly differ between the AIT and CT groups (95% CI, 0.85–1.72; *P* = 0.293).

### DCR

**Figure 9:**
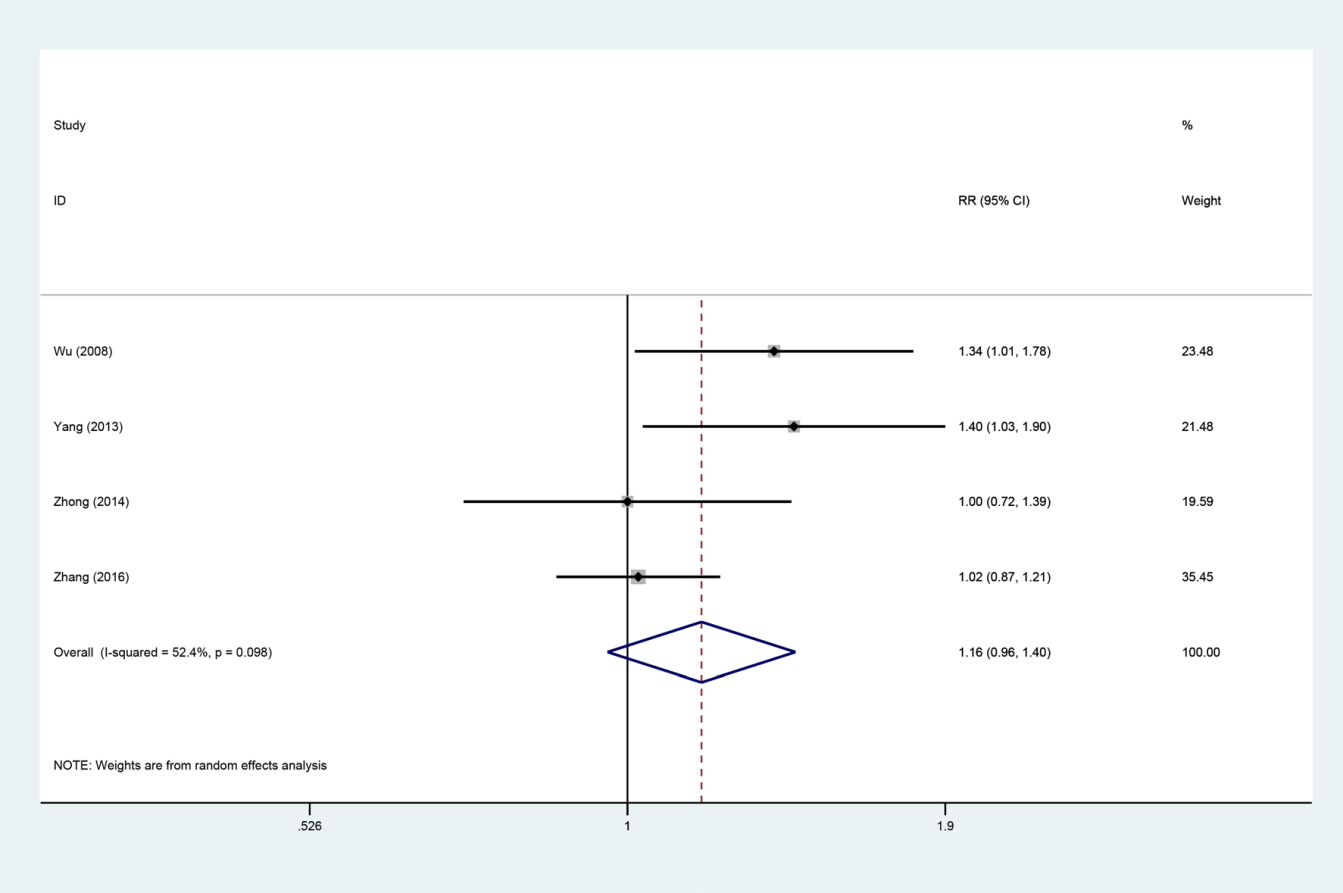
DCR between the AIT and CT groups

Four studies [9, 14, 15, 19], involving 323 patients (AIT group: *n* = 141; CT group: *n* = 182), were included in this analysis (Figure 9). High heterogeneity was observed (*P* = 0.098, I^2^ = 52.4%), and a random effects model was used. The disease control rate (DCR) did not significantly differ between the AIT and CT groups (95% CI, 0.96–1.40; *P* = 0.123).

### Sensitivity analysis

To evaluate the influence of single studies and analyze the effects of heterogeneity on the results, a sensitivity analysis was performed to determine the average relative risk (RR) in the absence of each study. If heterogeneity was found to be present, we would consider using the fixed-effects model.

With regard to 1-year OS, when 2 studies [13, 18] were omitted, the heterogeneity ranged from (P = 0.001, I^2^ = 62.9%) to (*P* = 0.38, I^2^ = 6%), whereas the combined RR ranged from 1.16 (95% CI, 1.06–1.26; *P* = 0.001) to 1.22 (95% CI, 1.14–1.30; *P* < 0.00001). With regard to 2-year OS, when 2 studies [8, 10] omitted, the heterogeneity ranged from (*P* = 0.099, I^2^ = 35.4%) to (*P* = 0.56, I^2^ = 0%), whereas the combined RR ranged from 1.38 (95% CI, 1.24–1.55; *P* < 0.001) to 1.44 (95% CI, 1.30–1.60; *P* < 0.00001). With regard to 5-year OS, when 2 studies [7, 12] were omitted, the heterogeneity ranged from (*P* = 0.001, I^2^ = 75.5%) to (*P* = 0.22, I^2^ = 32%), whereas the combined RR ranged from 1.56 (95% CI, 1.04–2.33; *P* = 0.032) to 1.66 (95% CI, 1.32–2.10; *P* < 0.001). With regard to 2-year PFS, when 1 study [18] was omitted, the heterogeneity ranged from (*P* = 0.033, I^2^ = 70.7%) to (*P* = 0.43, I^2^ = 0%), whereas the combined RR ranged from 1.53 (95% CI, 1.05–2.23; *P* = 0.029) to 1.31 (95% CI, 1.04–1.64; *P* = 0.02). In terms of the pooled DCR, when 1 study [19] was omitted, the heterogeneity ranged from (*P* = 0.098, I^2^ = 52.4%) to (*P* = 0.28, I2 = 21%), whereas the combined RR ranged from 1.16 (95% CI, 0.96–1.40; *P* = 0.123) to 1.27 (95% CI, 1.06–1.52; *P* = 0.01). As the heterogeneity could not be completely explained via sensitivity analysis, subgroup analyses were also conducted.

### Subgroup analysis

Subgroup analysis was conducted according to the effector cells used (LAK plus IL-2 versus CIK versus DC-CIK), number of cycles in the regimen (> 4 versus ≤ 4, the mean cycle length and total cycle length were considered to represent the same level), and nationality (China versus Japan). Subgroup analyses were only performed for 1-year OS and 2-year OS due to the availability of an adequate sample size (Table 4). CIK and DC/CIK significantly enhanced 1-year OS (95% CI, 1.16–1.59; 95% CI, 1.09–1.30) and 2-year OS (95% CI, 1.33–2.24; 95% CI, 1.10–1.44) compared with LAK plus IL-2 (95% CI, 0.86–1.21; 95% CI, 0.90–1.86). No specific difference was found in cycle or nationality.

**Table 4:** The outcome of subgroup analysis of AIT versus CT in relation of 1-year OS and 2-year OS

Group	1-year OS				2-year OS			
	No.of studies	RR (95% CI)	*P* heterogeneity	I^2^ (%)	No.of studies	RR (95% CI)	*P* heterogeneity	I^2^ (%)
**Total**	15	1.16 (1.06–1.26)	0.001	62.9	13	1.38 (1.23–1.55)	0.099	35.4
**Effector cell**								
Total	12				10			
LAK+IL-2	3	1.02 (0.86–1.21)	0.027	72.3	3	1.30 (0.90–1.86)	0.011	78.0
CIK	2	1.36 (1.16–1.59)	0.693	0	2	1.73 (1.33–2.24)	0.734	0
DC/CIK	7	1.19 (1.09–1.30)	0.751	0	5	1.26 (1.10–1.44)	0.471	0
**Cycle**								
Total	12				11			
> 4	8	1.18 (1.04–1.33)	< 0.001	75.5	7	1.52 (1.36–1.49)	0.939	0
≤ 4	4	1.19 (1.08–1.31)	0.376	3.4	4	1.28 (1.07–1.52)	0.305	17.5
**Nationality**								
Total	14							
China	10	1.20 (1.08–1.33)	0.096	39.3	7	1.41 (1.17–1.70)	0.166	34.4
Japan	4	1.08 (0.96–1.22)	0.010	73.5	5	1.36 (1.13–1.65)	0.046	58.8

### Publication bias

**Figure 10:**
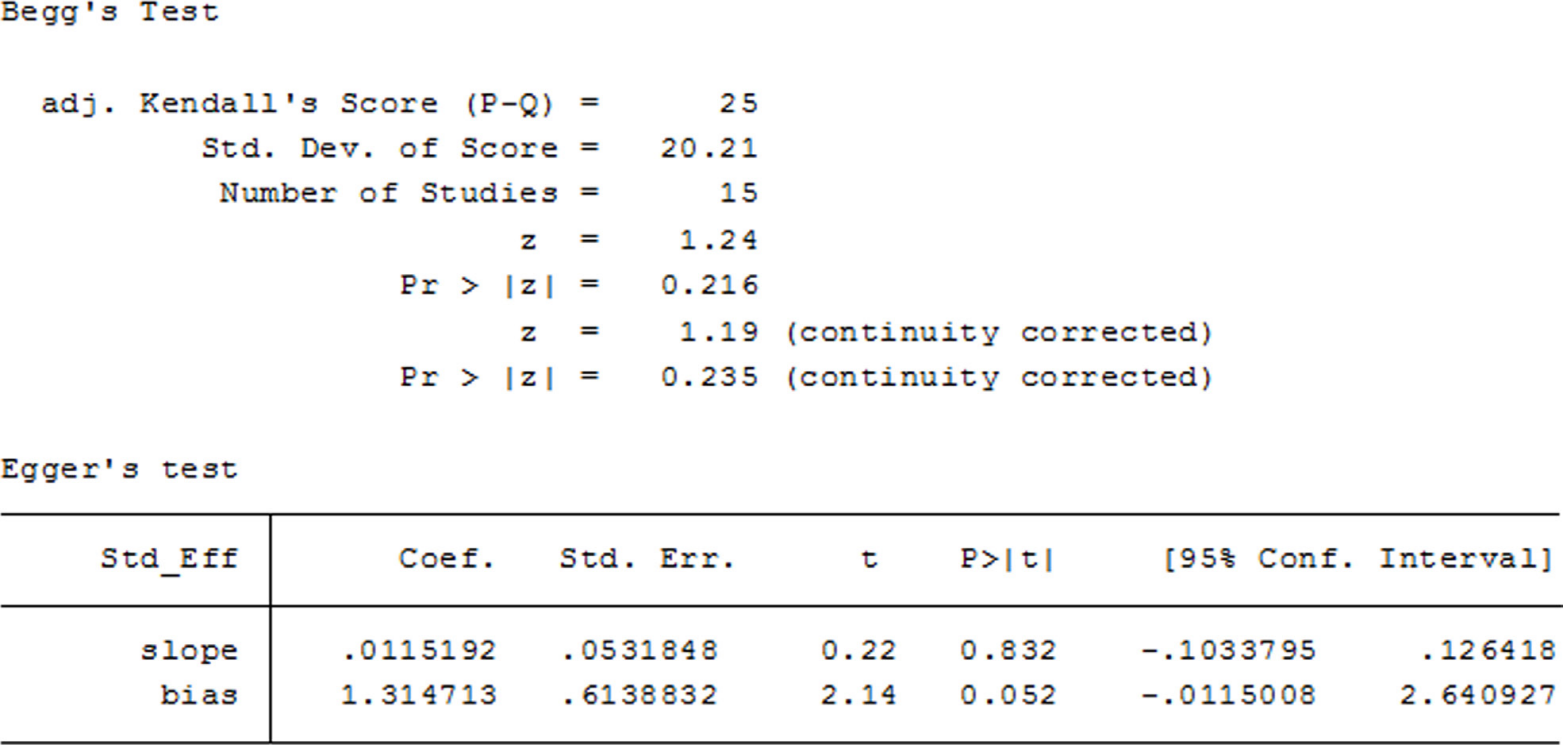
Begg's and Egger's test for 1-year OS

In the 15 included studies, there was no evidence of publication bias in terms of 1-year OS in NSCLC patients receiving AIT, as suggested by Begg's funnel plots test and Egger's regression test (Begg's *P* = 0.235; Egger's *P* = 0.052; Figure 10).

## DISCUSSION

In our meta-analysis, we evaluated the efficacy of AIT for NSCLC, particularly in terms of the OS, PFS, ORR, and DCR. The final results showed that AIT could significantly improve the OS and PFS, although it had a relatively minor effect on the ORR and DCR. In the subgroup analysis, DC/CIK and CIK rather than LAK plus IL-2 significantly improved the 1-year and 2-year OS, most of individual subgroup was consistent with the primary outcome. Publication bias and sensitivity analyses helped evaluate the heterogeneity between studies. Our research was meaningful because we inferred the prognosis of NSCLC patients with a stable condition from the overall outcome, and the OS and PFS were found to be significantly improved. AIT has major potential in clinical cancer treatment, which is an important characteristic of tumor immunotherapy, and the present study provides fundamental data based on which future tumor immunotherapy research can be conducted.

At present, immunotherapy is the fourth most common type of anti-tumor therapy, following surgery, radiotherapy, and chemotherapy. The European Organization of Research and Treatment of Cancer classified biological therapy of tumors as cytokine therapy, antibody therapy, vaccine therapy, and gene therapy [21]. The origins and effects of different effector cells differ. In particular, IL-2 is a cytokine that serves as a growth factor of all T cell subsets, and has a wide range of biological activities, including the promotion of B cell proliferation. IL-2 is also involved in immune responses, antibody reactions, and tumor immune surveillance [22]. High doses of IL-2 and other cytokines stimulate the peripheral blood lymphocytes to transform into LAK, which can kill tumor cells insensitive to NK [23]. TIL are infiltrating lymphocytes isolated from tumor tissue. Following intervention with IL-2 and CD3, TIL are activated and transformed into tumor-specific lymphocytes or NK [24]. CIK is a new type of immunocompetent cell that has been termed as an NK-like T (NK/T) lymphocyte due to their expression of CD3 and CD56 membrane proteins. CIK not only have the potent antitumor effects of T lymphocytes, but also possess the MHC-unrestricted cytotoxicity of NK [25]. Moreover, dendritic cells (DC) are potent antigen-presenting cells that can effectively resist the immune escape of tumor cells. Co-culturing tumor antigen-sensitized DC with CIK helps generate DC-activated CIK (DC/CIK), which promotes the maturation of DC and proliferation of CIK and increases the levels of cytokines (IFN, TNF, and CSF), without any side-effects, as those associated with CIK or MHC-unrestricted cytotoxicity in NK [26]. In fact, DC/CIK show a more profound anti-tumor effect, as compared with other effector cells, and are currently widely used in AIT for various cancers.

The administration of TIL in NSCLC patients helps prolong overall survival time duration to a greater extent, as compared to chemotherapy or radiotherapy, and 80% of patients do not report any severe adverse effects during treatment [6]. Recent studies showed that increasing the levels of CD8-positive TIL could represent a more potent treatment for melanoma [27]. In fact, CD137 may have the same role as CD8, and can help administrate TIL therapy for NSCLC [28]. γδ T cells regarded as another type of effector cell, belong to the T lymphocyte family, and account for approximately 5% of peripheral blood T cells. Antibody-expanded γδ T cells generating more effector T cells may release greater amounts of cytokine, and may immediately induce cytotoxicity functions [29]. Hanagiri [30] et al. stated that, when γδ T cells expressed specific αβ TCR molecules in addition to γδ TCR, they were more effective in NSCLC patients. NK are the innate effector cells of immune response to pathogens and cancer. Their phenotype is characterized by the expression of CD56 and an absence of CD3. Recent reports mentioned that targeted drugs, such as thalidomide or imatinib, could improve the levels of NK in the peripheral blood of NSCLC patients [31]. Moreover, CIK subsets contain regulatory T cells (Treg, CD4+ CD25+) that can suppress the immune function of the tumor. DC/CIK down-regulate the inhibitory effect of Treg on the immune system of patients by reducing the levels of Treg in the CIK subsets. Furthermore, Zhao [32] et al. found that the average Th2 cytokine (IL-4, IL-10) levels were higher in NSCLC patients before immunotherapy, and hence, DC/CIK could overcome the dominant status of Th2 cytokines and up-regulate the anti-tumor effect. Moreover, the researchers found that DC/CIK induced rare side-effects in combination with chemotherapy. Shi [33] et al. believed that the synergistic anti-tumor efficacy of DC/CIK involved the normalization of the tumor vasculature and reduction in the hypoxic area in the tumor microenvironment.

An increasing number of studies are focusing on the tumor micro-environment. In particular, CTLA-4, PD-1, and PD-L1 blockade exhibited clinical benefits and good tolerance, with limited adverse effects [34, 35], although the actual effectiveness needs to be confirmed. Thus, it appears that AIT is effective and has limited side-effects, and is hence a useful treatment.

Zeng [36] included 4 RCTs in their small meta-analysis on the OS and adverse effects of AIT in NSCLC patients; in contrast, we included 15 high-quality studies in our meta-analysis, reported a more useful prognostic outcome, and provided a comprehensive and detailed description of the potential underlying AIT mechanism.

We found that DC/CIK yielded greater benefits and had a more potent cytotoxic function as compared with LAK in combination with IL-2 in subgroup analysis. As a result of the reduced side-effects, we believe that DC/CIK vaccines can be considered in clinical practice, which may improve patient prognosis and quality of life. However, due to the strict culture conditions and time-reliance inducing restrictions of DC/CIK, the cost of this treatment should be carefully reviewed and controlled prior to practical application. Consequently, further studies are needed to estimate the efficacy of the various treatment strategies for NSCLC.

The results of our sensitivity and subgroup analyses were similar and robust. Our conclusion regarding most of the parameters (except DCR) was not significantly modified after excluding studies with high heterogeneity, and after conducting LAK plus IL-2 and DC/CIK strategies analysis. High heterogeneity was observed for 1-year OS, 2-year OS, 5-year OS, 2-year PFS, and DCR, and the main reasons for the heterogeneity included differences in drug regimens and individual differences. An insufficient number of patients was present in the high heterogeneity studies, and almost all these studies demonstrated a significant inverse association. Furthermore, compared with individual studies, the characteristics of studies design, clinical settings and patients differed in various aspects. For instance, the earliest research was conducted in 1995, whereas the latest study was conducted in 2016; during this period, marked progress had been made in detection technology. Some studies involved AIT with LAK in combination with IL-2, whereas the recent studies use DC/CIK. In addition to the variables studied in the previous study, other factors such as the patient population, tumor stage, type of pre-treatment, dosage and duration of AIT, treatment of the CT group, race differences, regional differences, and individual difference could lead to heterogeneity in the present study. However, the inclusion of 15 studies involving 1684 participants in our meta-analysis strengthened our ability to determine a significant association and provide a more reliable evaluation of AIT.

The present meta-analysis has certain limitations. First, the inclusion of additional studies led to an increase in the heterogeneity. Some indicators exhibited marked heterogeneity, which was inevitable due to the various factors involved. However, the heterogeneity has been explained in the meta-analysis. Second, the AIT cycles were not uniform, we could not determine the appropriate duration of AIT to maximize its effectiveness; this inhibits its application in clinical practice. Moreover, the reduced adverse effects with AIT were reported in only 4 trials [10, 15, 17, 19] (Table 5), the pooled result from these little sample sizes showed AIT had acceptable or even less toxicity compared with CT. Third, we did not include an adequate number of studies to yield credible results, and most of the studies were based on an Asian population, which could affect the final conclusion. Fourth, in 2 studies [8, 19], a random sequence of inclusion was not used as the patients could not afford AIT; in those cases, the researchers allowed the patients to choose appropriate therapies based on their circumstances. Finally, though there was no significant difference in clinical stage, histology, pre-treatment et al., these discordant elements might hinder us to draw final conclusion, we still needed more RCTs with consistent patients’ conditions to help interpret our results. All the included studies only concentrated on AIT in combination with chemotherapy or radiotherapy versus CT, and hence, it was difficult to estimate the efficacy of AIT alone. The efficacy of AIT was associated with patient age, race, smoking, alcoholism and heredity; therefore, further stratified analyses are needed on these indicators in the future. Due to discordance of AIT and CT, it is a future direction to compare specific AIT (LAK or TIL or DC/CIK) to specific CT (chemotherapy or radiotherapy or no adjuvant treatment (placebo)).

**Table 5:** Adverse effects of AIT reported in 4 included studies

Adverse Events	AdoptiveImmunotherapy (events/total)	Control Therapy (events/total)	RR (95% CI)	*P* value	Heterogeneity	
					I^2^ (%)	*P* value
Fever [10, 17, 19]	17/91	44/129	0.75 (0.22–2.54)	0.650	75.0	0.020
Anemia [10, 15, 17]	13/70	20/68	0.65 (0.36–1.15)	0.140	0	0.770
Leucopenia [10, 15]	53/72	63/72	0.84 (0.71–0.99)	0.040	38.0	0.200
Nausea [10, 15, 19]	35/93	56/133	0.71 (0.54–0.92)	0.010	0	0.840
Rash [10, 15, 17]	28/100	60/98	0.44 (0.17–1.16)	0.100	84.0	0.002
Fatigue [10]	3/42	24/42	NA^a^	NA	NA	NA
Diarrhea [17]	9/28	6/26	NA	NA	NA	NA
Thrombocytopenia [15]	3/30	4/30	NA	NA	NA	NA
Anorexia [19]	3/21	6/61	NA	NA	NA	NA
Allergy [19]	1/21	0/61	NA	NA	NA	NA
Myelosuppression [19]	2/21	8/61	NA	NA	NA	NA
Radiation pneumonitis [19]	7/21	11/61	NA	NA	NA	NA

^a^no available statistical data.

In conclusion, we observe that AIT has a significant role in NSCLC and the tolerability can be improved in AIT regimen. Even so additional studies with a large sample and high-quality RCTs are needed to validate these findings.

## MATERIALS AND METHODS

This systematic review and meta-analysis was conducted in accordance with the PRISMA guidelines (Supplementary Table 1) and has been registered in PROSPERO (CRD42017060172).

### Search strategy

Parallel randomized controlled trials (RCTs) and high-quality observational studies that compared AIT with control therapies (CT) in NSCLC patients were collected. We systematically searched PubMed, the Cochrane Library, EMBASE, Medline, and Web of Science, from the time of inception of each database to February 31, 2017, without any language restrictions. We used the following combined text and MeSH terms: “Carcinoma, Non-Small Cell Lung” and “Immunotherapy, Adoptive Cellular”. The complete search terms for Pubmed included: (Carcinoma, Non-Small-Cell Lung [MeSH Terms] OR Carcinoma, Non Small Cell Lung [Text Word] OR Carcinomas, Non-Small-Cell Lung [Text Word] OR Lung Carcinoma, Non-Small-Cell [Text Word] OR Lung Carcinomas, Non-Small-Cell [Text Word] OR Non-Small-Cell Lung Carcinomas [Text Word] AND (Immunotherapy, Adoptive Cellular [MeSH Terms] OR Adoptive Immunotherapy [Text Word] OR Adoptive Immunotherapies [Text Word] OR Immunotherapies, Adoptive [Text Word] OR Cellular Immunotherapy, Adoptive [Text Word]). We collected the studies in accordance with the requirements in various possible ways.

### Study selection

We included studies that met all the following conditions: (a) Patients: adult patients with NSCLC diagnosed via imaging, pathology examination, or other adjuvant diagnosis based on the WHO criteria. Patient inclusion was not restricted based on sex, race, or nationality, and pre-treatment (surgery, chemotherapy, or radiotherapy) was allowed to ensure that they were suitable candidates for immunotherapy; (b) Intervention: AIT or AIT plus adjuvant therapy; (c) Comparison: chemotherapy, radiotherapy, adjuvant methods, plus different doses/durations of immunotherapy, or placebo; (d) Outcome: OS or progression-free survival rate (PFS) or the objective response rate (ORR) or disease control rate (DCR); (e) Design: RCTs and observational studies (prospective or retrospective cohort studies). The proportion of cases that were lost to follow-up, that withdrew from the study, or that encountered unexpected conditions did not exceed 20%. The patients’ clinical stage, histology, sex, age, pre-treatment existed no significant difference and was comparable among groups.

The most complete and novel reports were included for data extraction and assessment, if the objects were duplicated. Reviews without original data, case reports, meta-analyses, letters, expert opinions, and animal studies were excluded.

### Data extraction

Two independent investigators reviewed the research titles and abstracts, and the eligible studies were then retrieved for full-text assessments. The assessments of exposure and outcome, duration of follow-up, and statistical control for potential confounding factors were conducted by 2 investigators via consensus; disagreements were resolved by a third investigator.

We extracted the following useful data from the studies: total number of participants, region, immunotherapy, control therapies, duration. We then estimated the OS, PFS, ORR, and DCR of the patients, which was pooled through relative risk (RR). If these indicators could not be directly obtained, we inferred the values from the individual data curves presented in the studies. We also reviewed the adverse effects of AIT specified in the included studies.

### Quality assessments

Two reviewers assessed the risk of corresponding bias using the “Cochrane risk of bias tool” for each RCT. Observational studies were evaluated using the Newcastle-Ottawa Scale (NOS) [20]. Moreover, the reporting and ascertainment of included strains, the statistical power, and the funding and potential for conflict of interest associated with individual trials were assessed.

### Statistical analysis

We evaluated the efficacy of AIT based on the following 4 indicators: OS, PFS, ORR, and DCR. The RR and 95% CI of all the indicators were recorded; the hazard ratios (HR) and incidence rate ratios were directly considered as the RR. The homogeneity of the effect size across studies was tested using Q statistics (at the *P* < 0.10 level of significance). We also calculated the I^2^ statistic to help assess heterogeneity (high heterogeneity > 50%; low heterogeneity, < 50%). Data were analyzed using fixed-effects models when *P* > 0.10 for the Q statistic; in other cases, random-effects models were used [37]. For the meta-analysis of each outcome, we conducted pre-planned sensitivity analyses restricted to trials that included the efficacy of AIT. We also conducted pre-specified subgroup analyses based on the type of effector cells used, patient nationality, cycles of AIT, and the effect of these variables on outcome.

The presence of potential publication bias was assessed using Begg's funnel plots test [38] and Egger's regression test [39]. All statistical analyses were performed with Stata 12.0; a *P* value < 0.05 was considered to be significant, except where otherwise specified.

## SUPPLEMENTARY MATERIALS AND TABLES





## Abbreviations

AIT: Adoptive immunotherapy
CT: Control therapy
NSCLC: Non-small cell lung cancer
RCTs: Randomised controlled trials
OS: Overall survival rate
PFS: Progression-free survival rate
ORR: Objective response rate
DCR: Disease control rate
RR: Relative risk
95%CI: 95% Confidence intervals
NK: Natural killer cells
CTL: Cytotoxic T lymphocytes
LAK: Lymphocytes activated killer cells
CIK: Cytokine-induced killer cells
DC: Dentritic cells
TIL: Tumor infiltrating lymphocytes
NKT: NK like lymphocytes
AKT: Activated killer T cells
PRISMA: Preferred Reporting Items for Systematic Review and Meta-Analysis
PROSPERO: International Prospective Register of Systematic Reviews
IL-2: Interleukin 2
IFN: Interferon
CSF: Colony stimulating factor
PD-1: Programmed death 1
PD-L1: Programmed death-ligand 1
CTLA-4: Cytotoxic T lymphocyte-associated antigen-4
