# Supplementary material for: Adoptive immunotherapy shows encouraging benefit on non-small cell lung cancer: a systematic review and meta-analysis
Source: Oncotarget. 2017 Jul 19;8(68):113105–19. doi: 10.18632/oncotarget.19373 (PMC5762575; doi:10.18632/oncotarget.19373)
Supplement: Supplementary file 1 [file oncotarget-08-113105-s001.pdf]

Adoptive immunotherapy shows encouraging benefit on non-small cell lung cancer: a systematic review and meta-analysis

SUPPLEMENTARY MATERIALS

Supplementary Table 1: PRISMA 2009 Checklist. See Supplementary_Table _1
